# Supplementary material for: Representational capacity shapes both the “whether” and “how” of social learning
Source: PLoS Comput Biol. 2026 Jul 15;22(7):e1014504. doi: 10.1371/journal.pcbi.1014504 (PMC13384400; doi:10.1371/journal.pcbi.1014504)
Supplement: Attachment — Submitted filename: response-letter.pdf [file pcbi.1014504.s003.pdf]

Authors' Response to Reviews of

Representational(/cognitive) capacity shapes both the "whether" and "how" of social learning

Anonymous authors
,

RC: ***Reviewer's Comment***, AR: Authors' Response, ☐ Manuscript Text

We would like to thank both reviewers wholeheartedly for their thoughtful and thorough engagement with our work; we feel the manuscript is considerably improved following their feedback. The main changes made are:

1. New results added for a simpler action-imitation social learning model
2. A more cautious framing of our particular capacity implementation (e.g. "cognitive capacity"→"representational capacity")
3. Better contextualisation of our model with respect to relevant existing work
4. An expanded limitations section within the discussion
5. Results figures made simpler and more consistent where possible

Please see below for our point-by-point responses, and descriptions of the changes that have been made.

1. Reviewer #1

RC: ***This paper presents a series of simulation experiments investigating the effects of cognitive capacity and social learning strategy on the evolution of agents in a simple learning and foraging task. In the task, agents receive partial information about the environment and the preferences of other agents, and decide between eating or discarding the current mushroom. An underlying rule determines the reward outcomes based on N binary features of the mushroom. Agent fitness is tracked: agents die if they fall below a fitness threshold (or periodically with some probability), and are replaced probabilistically with high-fitness agents. In a series of four simulated experiments, the authors investigate the relative abundance of learning strategies within the population as a function of time and "cognitive capacity", which quantifies agent's ability to retain information gathered from the environment in their subsequent learning and decision making.***

RC: ***The authors show that both cognitive capacity and social learning strategy have strong effects on the evolution of the population. They begin by comparing asocial learning to unbiased social learning, in which agents randomly query other agents to acquire additional social information. Even in this simple case, where only one social learner is present amongst a population of asocial learners, they show an unexpected non-monotonic relationship between capacity and the benefits of social information use. In subsequent experiments, the authors expand on these findings by stimulating additional evolutionary dynamics, a wider range of social strategies, and more detailed analyses about the resulting population breakdown and/or social exchange. They conclude by drawing several insights about the value of social***

exchange relative to the complexity of the environment, and how the relative effectiveness of social learning strategies shifts over time and environmental parameters.

RC: ***I thought this paper was clear, concise, and persuasive. The authors positioned their contribution effectively within the broader field of social learning, arguing that previous studies have failed to look at agent's information processing capacity and of symmetric constraints in both learners and providers of information. While I am not familiar with these fields, both of these features seem highly relevant in studying social learning in any naturalistic task or when dealing with any realistic cognitive system. The methods section was fairly straightforward, and included the key details necessary to understand the simulated agents and experiments, with only a few points of confusion. Similarly, the four experiments presented in the paper followed a logical progression, from establishing a baseline of behavior and analysis, through the core experiments establishing the U-shaped dependence on capacity, and ending in additional analyses that supported their explanations for the emergence and dominance of certain social learning strategies. The figures were clearly laid out, using colors to effectively communicate a variety of complex relationships. I appreciated the two choices of 'complex' social learning strategies, but felt that the intuition and mechanics of these agents could have been elaborated in greater detail (see below). I found the relative success of the four agent types to be one of the most interesting aspects of the paper, and the additional analyses in Fig 5C and 6 seemed to support the author's story about the added benefits of social information in each case. The inverse-U shaped relationships are also interesting, and showcase the utility of using computational modelling to investigate multi-agent social systems, where intuitions can often fail.***

AR: We thank the reviewer for these kind words!

RC: ***I had a few minor complaints and suggestions, which I will list here:***

RC: ***The term "cognitive capacity" strikes me as too general, and risks overinflating the (real) value of the paper. The authors clearly note that "There are multiple ways we could achieve this within the TS framework, and we report results for different variants in the SI." I appreciate this anticipation and engagement with the question, and the SI results do offer some additional insights into how cognitive capacity might be realized in this framework. The SI results also show that the main results hold under these different realizations, although with some variations that might be worth exploring in greater detail. Furthermore, the authors provide citations of similar work that "uses information bottleneck as a principled way to model capacity limits". Nonetheless, as a cognitive scientist who studies the ability of simulated biological systems to implement abstract cognitive algorithms (like the ones employed here), I still feel that "cognitive capacity" is too big an umbrella for the manipulations being done here. I would be more comfortable with a term like "representational capacity", which emphasizes that the limitations are on how much information the agents can store (or use) accurately, rather than how they can use it. Representational capacity applies both to zeroing out individual features (the main manipulation), to noise in feedback (SI 1.2), and, to a lesser extent, to imperfect memory (SI 1.1). Representational capacity can be contrasted with "functional capacity", which emphasises the inability for an agent to compute certain classes of functions entirely. An example of functional capacity might be limitations on agent's rules for forming or updating belief distributions, their ability to identify the fittest individual, etc. Whatever language the authors decide to use, I would advise them to make limited claims about how broadly their manipulations capture cognitive capacity in intelligent animals.***

AR: This is a fair point; we accept that the initial version of the manuscript made too general a framing of our capacity model. To address this, we have removed the specific phrasing "cognitive capacity" from both the title and body of the manuscript, replacing it variously with "representational capacity", "information-processing capacity", or in places simply "capacity". We have also updated the Methods section to be more explicit

about what our particular implementation is capturing:

> Since our goal is to study the impact of capacity constraints, we must somehow limit agents' ability to process information. There are multiple ways we could approach this within the TS framework; we do so in terms of the *number of features* (in x_t) that agents can perceive during each trial. This choice of implementation is motivated by a focus on *representational* capacity: that is, agents' ability to represent the task-relevant features of their environment. This is distinct from e.g. functional capacity—regardless of how much information is retained from x_t, all agents are equally capable of performing the computations necessary to evaluate rewards, update beliefs, select actions, etc.

RC: ***They might also devote some space in the discussion to contextualizing this choice relative to broader notions of social intelligence and the complexity of social exchange.***

AR: Reviewer 2 raised a similar point, and we agree that the lack of consideration of the costs involved in executing our social learning models is an important limitation. We thank both reviewers for pressing us on it, and have now dedicated two paragraphs to it in the discussion (lines 650-683 in the revised manuscript).

RC: ***Similarly, I'd appreciate a bit more qualitative discussion about the choice to "model social learning as an additional belief update ... that takes place before choice", and the choice to model social cues as an expressed belief about edibility. For example, how might the experiments and results change if agents communicated their underlying feature-based belief structures directly?***

AR: Following feedback from Reviewer 2 (see below), we have now added an additional variant of the agent model that uses simpler 'policy-based' social learning (i.e. copies actions directly). The methods section now frames these two variants as capturing distinct levels of the social learning 'hierarchy'; we feel that this better makes the case for our social-belief-update approach. Regarding the way we model social cues: having agents communicate their full underlying beliefs directly is an interesting idea. Without running new experiments, our intuition is that it would increase the value of social learning across all levels of capacity (and so would probably not drastically change the overall inverse-U trend). However, we feel that doing things this way could introduce some issues for how the model is interpreted:

1. It feels like it would move us closer to explicit teaching/instruction (which would be very interesting! But probably best approached separately from the current work).
2. It could clash with the information bottleneck framing—communicating full beliefs would be much 'higher-bandwidth' than the current binary cue. To make this realistic, we would need to add some additional bottleneck that acts as a lossy channel for belief transmission; but this would add more complexity to a model that already has quite a lot of moving parts (and in a sense the current cue setup can be seen as a rough approximation of this).

RC: ***Equations 8 and 9 for the conformist and fittest social learners need additional elaboration. A few sentences to describe the high-level logic of those learning strategies, and why the particular mathematical forms were chosen to implement this logic, would go a long way.***

AR: We have rewritten Section 3.3 to make it clearer what these social learning strategies are doing, and how the specific mathematical expressions capture that. We have also added citations to additional prior work that helps contextualise why these two particular strategies are of interest.

RC: ***Fig 5a, why do conformist and copy-fittest make up such a small proportion of the final population across a wide range of capacities? Given Fig 5C, shouldn't these two strategies generally acquire more correct***

social information, and subsequently out-compete the naive or asocial learners? This is also the trend I expected given Fig 6B: unbiased learners and asocial learners tend to die out over time. Fig 5A thus seems to contradict one of the central conclusions "Specifically, we found that learners who selectively use cues from only the fittest agents dominated lower-capacity populations". Perhaps the n=4 environment was an outlier in this case?

AR: We thank the reviewer for highlighting this issue. We accept that our original language ("dominated") was misleading, and overstated the specific claim we wish to make—which concerns the relative adaptiveness of conformist vs success-biased social learning, and not whether strategic social learners drive non-strategic or asocial learners to extinction. We have now softened this language, e.g.

> Specifically, we found that learners who selectively use ~~cues~~ information from only the fittest agents ~~dominated~~ performed well in lower-capacity populations, whereas conformist learners that simply followed the majority opinion were most successful in higher-capacity populations.

As to why the curves in Figure 5C don't translate to higher strategic proportions in 5A: these reliability values, while useful for guiding intuition, nevertheless represent idealised estimates obtained under conditions where there was no *actual* social learning taking place. The two sets of results are therefore not 1:1 comparable—for example, the proportions in 5A will presumably be subject to frequency-dependent effects, where e.g. effective social learning requires a certain level of asocial exploration to be present within the population (i.e. Roger's paradox). We also note that the simulations in experiment 4 (Figure 6) were run for $10\times$ as many timesteps (10^5 vs 10^4), which might be expected to produce more uneven final proportions. Unfortunately, running the experiment 3 simulations for 10^5 timesteps was not computationally feasible, since we had to run a large number of independent simulations over different capacity values (which we did not have to do for experiment 4 due to co-evolving rather than imposing κ).

RC: ***Overall, this is an excellent paper that I can happily endorse for publication, with only a few minor edits for clarity and contextualization.***

AR: We thank the reviewer again for their generous words, and their contribution to what we believe is now a stronger paper!

2. Reviewer #2

RC: ***This manuscript presents what I found to be a very interesting investigation of how cognitive capacity influences social learning. Through a series of simulations, the author presents findings showing how cognitive capacities influence the emergence of social learning strategies, which can shift from success-biased to conformity-biased depending on capacity. I think these findings are of great interest to a wide readership, but in particular for the field of cultural evolution, which is increasingly interested in more cognitively sophisticated models of social learning.***

RC: ***Overall, I found the paper to be quite well written, although I have a number of minor clarification issues I address at the bottom. In addition, I also identified some relatively more major issues, which I address now in more detail.***

AR: We thank the reviewer for these kind words!

2.1. Cognitive costs of social learning

RC: ***Here the authors focus on a setting where cognitive capacity only influences asocial learning. However, different kinds of social learning also come with their associated cognitive costs (Wu, Velez & Cushman, 2022). Simple imitation may be quite cheap (as is assumed in these simulations), but more sophisticated forms of social learning, such as Theory of Mind inference, are arguably even more complex than asocial learning. Thus, decreasing capacity doesn't necessarily favor social learning, since social learning also comes with cognitive costs. I think this could be clarified in the paper.***

AR: Reviewer 1 raised a similar point, and we agree that the lack of consideration of the costs involved in executing our social learning models is an important limitation. We thank both reviewers for pressing us on it, and have now dedicated two paragraphs to it in the discussion (lines 650-683 in the revised manuscript). We would also emphasise here that our results are consistent with decreasing capacity not necessarily favouring social learning; indeed we find social learning is less useful at low than at moderate capacity due to the quality of social information available.

RC: ***Additionally, I think there are some idiosyncrasies in how social learning is implemented here that need to be unpacked in greater detail. Line 262 states that the focus is on the initial emergence (in evolutionary timescales) of social information, which is used to justify an initial focus on indiscriminate social learning. However, if that is the case, why do you need the rather complex Eq. 6 whereby an expectation of reward is emitted by each peer, which is then binarized in a somewhat arbitrary fashion. Wouldn't it be more straightforward to just implement frequency dependent copying? Or some form of policy-based social learning rather than a more complex value-based strategy (cf. hierarchy from Wu, Velez & Cushman, 2022)? Surely biased social learning would emerge before the kind of social learning represented in Eq. 6? Perhaps a better justification could be provided or the limitations of these assumptions mentioned more explicitly in the discussion.***

AR: This is a fair criticism. Our original rationale for the cue/belief approach was to try and capture a more general space of social learning behaviours than just action imitation, and to allow social and asocial learning to coexist within a single individual. On reflection, we agree that this may conflict with our "initial emergence" framing. To that end, we have implemented additional variants of all main-text simulations using direct action imitation, and now report results for both variants. While there are some differences between the two (which we point to in the text without spending too much time on them), our main qualitative findings were unaffected.

2.2. Cognitive capacity via corrupting reward encodings vs. effortful uncertainty directed explanation

RC: ***I thought the current implementation of capacity was quite innovative (Eq. 4). However, I also found there to be rather limited connections to previous work in resource rational (asocial) learning. The Lai & Gershman work is applied to policy complexity and Zaslavsky applies it to naming conventions. Of course it's justifiable to introduce this as a new mechanism, however it also feels like a missed opportunity to connect to more well-studied mechanisms of asocial learning that are associated with cognitive costs. For instance, by connecting to directed exploration as being an aspect of asocial learning is specifically sensitive to reduced cognitive capacity, be it from working memory load (Cogliati-Dezza, Cleeremans, & Alexander, 2019) or time pressure (Wu, Schulz, Pleskac & Speekenbrink, 2022). This could be implemented using UCB sampling, where more limited agents would have lower information bonuses for exploration. I'm not insisting on this also being added to the simulations, but some connection to the cognitive costs of exploration could help this work reach a broader audience.***

AR: We thank the reviewer for bringing this line of work to our attention. We have not run new simulations with

the UCB idea, having decided instead to prioritise doing so for the simple-imitation social learning variant. We have however added a paragraph to the discussion section (lines 629-649 in the revised manuscript) that mentions these and other results concerning the effect of resource constraints on asocial learning, and suggests integrating richer forms of exploration as a promising avenue for future work.

2.3. Difficulty in interpreting the figures

RC: ***I found some of the results very hard to read from the figures. This was particularly the case for Figure 4, where even after spending a lot of time with it, I still don't think I really understand what the red line and the green bars represent. In addition, plots showing related results present them in entirely different formats, making it hard to compare findings from one set of simulations to the next.***

AR: We apologise for these difficulties in parsing the figures. We have made the following changes to Figures 3-6, which we hope makes them more consistent and easier to follow:

1. **Figure 3:** we have changed the x-axis from absolute to relative capacity ($\kappa \to \kappa/n$) for consistency with Figures 4 and 5
2. **Figure 4:** we have abandoned the barplot format, and instead adopted the same format and styling of Figure 3. Rather than asking the reader to mentally subtract the red-line baseline proportion from the bar heights, we now give the y-axis as difference in proportion between "real" and baseline/neutral-drift simulations—a small diagram in the top-right corner of the figure illustrates how this is computed. We have also added additional panels that show the same data in heatmap form, in case the line plots are still difficult to tease out.
3. **Figure 5:** we have replaced the stacked-bar plots with continuous stacked-area plots, to avoid any strobe-like effect.
4. **Figure 6:** without removing the proportions-over-time lineplots, we have added a stacked-area plot of the same data, for consistency/ease of comparison with Figure 5. We have also added markers at the left edge of the lineplot to highlight the initial proportion of each type. Finally, we discovered that an error in our plotting code was incorrectly normalising these proportions, leading to y-axis values in the lineplots that summed to > 1—we apologise for this error and have now fixed it.

RC: ***Additionally, I was also wondering why no strategy ever dominates in the simulations. Is this potentially suggesting they haven't been run for enough iterations? What implications would there be for this lack of dominance of a single strategy? Would we expect a proportion of the population to be incapable of social learning? Or would we expect some degree of specialization?***

AR: These are very interesting questions. First, it is certainly possible (even likely) that we would see more pronounced differences between strategy proportions if simulations were run for longer. Computational constraints unfortunately made doing so impractical—but we also feel that the value in our simulation results lies in demonstrating how relative selection changes as a function of κ and n; we are less concerned with being able to claim that a particular strategy achieves fixation under particular conditions. Second, frequency-dependent effects mean that we probably shouldn't expect social learning to dominate even under conditions that favour it (e.g. Roger's paradox, as pointed out in the reviewer's first minor comment). Whether or not this should be interpreted explicitly in terms of specialisation, we are unsure—what we would say is that our results are certainly consistent with mixed populations in which different learning strategies are able to coexist. Finally, on a more practical level, our simulation dynamics are set up in such a way that ongoing mutation allows non-dominant types to persist or re-enter the population.

2.4. Minor comments

RC: ***Line 33-34 "critical mass". Isn't the issue usually the opposite? There's high frequency dependent fitness for being a social learner when the population is full of asocial learners, but low fitness when the population is saturated with social learners (Rogers, 1988). Thus, it's easy for social learning to invade, but difficult for it to dominate.***

AR: We agree with the reviewer that the original "critical mass" phrasing, though intending to refer to more CCE-like benefits of widespread social transmission, was misleading. We have removed that passage from the introduction.

RC: ***Line 83: Barkoczi & Galesic (2018) address this question. Although it's still true it's been less studied***

AR: We thank the reviewer for making us aware of Barkoczi & Galesic's work, and have added a citation to it in this passage (lines 100-101 in the revised manuscript).

RC: ***Fig. 1. More labels are needed since the figures are not very self-explanatory. Panel c needs axis labels and the n variable should perhaps be explained***

AR: We apologise for the difficulty in interpreting Figure 1. We have added more explanatory text to the figure itself, and have updated component A with an illustration of what n means.

RC: ***Line 118. This seems similar to the findings from Roberts-Gaal, Bolic, & Cushman (2025)***

AR: We thank the reviewer for highlighting this connection, and have added mention of it to the discussion (lines 620-627 in the revised manuscript).

RC: ***Line 124. There are multiple definitions of contextual bandit. Maybe cite a similar paradigm for clarity.***

AR: We have added a citation to Schulz, Konstantinidis, & Speekenbrink (2018), which uses a very similar version of the contextual bandit problem (although their rewards are stochastic while ours are deterministic).

RC: ***Reward function. The manuscript uses a rule-based reward function. What idiosyncrasies of rule-based generalization are being captured here vs. alternative accounts of similarity-based generalization (see Wu, Meder & Schulz, 2025 for a recent review)? Would this potentially influence the social vs. asocial trade-off? Perhaps this could be mentioned in the discussion***

AR: This is a great question. Speculating, the asocial learning ability of agents using continuous-similarity representations (rather than symbolic rule hypotheses) may fall off less dramatically at low capacity—further reducing the usefulness of social learning in this range, and magnifying the maladaptive effect that we currently only observe in low-complexity environments. We have added a short paragraph to this effect to the discussion (lines 684-700 in the revised manuscript).

RC: ***Line 172. Perhaps clarify that this is the asocial strategy***

AR: We have updated the subsection heading to clarify this ("Thompson Sampling" $\rightarrow$ "Asocial learning as Thompson Sampling")

RC: ***Line 184 is separated by an entire paragraph before the related equation at the top of Eq. 4 is presented. This maybe it a bit hard to follow the methods***

AR: To make things easier to follow here, we now give the action-selection equations first in terms of the uncompressed features x_t, immediately after the verbal description (line 201 in the revised manuscript). We then re-present it in terms of $z_t^{(m)}$ in the following paragraph (line 214), after describing how we introduce

capacity limits.

RC: ***Line 228. Why is this the case? Is it because of noisy exploration induced by noisy representations?***

AR: Good question. It is indeed a result of noisy exploration, but this is not induced by noisy representations of features (if that is what the reviewer meant here; apologies if not). At $\kappa = n$ the agents have perfect noise-free access to environment features—so agents that survive long enough to learn the true rule will settle into perfect behaviour, reaching (and staying at) h_{max}. But at $n = 6$ the search space is so enormous that many agents will die as a result of unlucky random exploration before this point. Therefore the *average* fitness across multiple independent runs (as is shown in Figure 2) will be below h_{max}.

RC: ***Fig. 3 please use a continuous color scale for n to make it easier to read. Also try to avoid a stroop effect where some of the n-colors also accidentally align with the top and lower part of the figure (adaptive vs. maladaptive) that may mislead the reader***

AR: This has now been fixed. We have also updated Figure 3 to use relative capacity (κ/n) on the x-axis, to make it more consistent with later Figures.

RC: ***Line 338. Why does it disappear?***

AR: The likely explanation for this is fairly simple: at low-capacity, social cues are very error-prone, and so may actually push the social learner's beliefs *away* from the true rule. In low-complexity environments, the asocial learning problem is tractable enough that these cues can therefore do more harm than good, and so social learning becomes maladaptive. In higher-complexity environments, at the values of capacity where this effect might appear, asocial learners are struggling so much that updating on unreliable cues makes very little difference. We have added a very brief sentence to this effect in the manuscript text (line 388 in the revised manuscript).

RC: ***Line 353. How can we verify this? These are entirely different figures***

AR: We have revised the language we use here:

> As Figure 3 shows, we ~~reproduce the central relationship found in~~ observe a similar qualitative pattern to that found in Experiment 1: selection for social learners varies as an inverse-U-shaped function of capacity; and is stronger in more complex environments (higher n).

In addition, we hope that the changes made to Figure 3 will make it easier to compare the results of Experiments 1 and 2.

RC: ***Fig. 5. Why does no strategy dominate?***

AR: We have addressed this under Section 2.3 above.
