# Supplementary material for: Representational capacity shapes both the “whether” and “how” of social learning
Source: PLoS Comput Biol. 2026 Jul 15;22(7):e1014504. doi: 10.1371/journal.pcbi.1014504 (PMC13384400; doi:10.1371/journal.pcbi.1014504)
Supplement: Attachment — Submitted filename: response-letter_auresp_2.pdf [file pcbi.1014504.s004.pdf]

Authors' Response to Reviews of

Representational capacity shapes both the "whether" and "how" of social learning

Anonymous authors
,

RC: ***Reviewer's Comment***, AR: Authors' Response, ☐ Manuscript Text

We wish to once again thank both reviewers for their feedback. We are glad that the previous round of revisions addressed many of their concerns; we hope that the additional changes we detail here will satisfy those that remain.

1. Reviewer #1

RC: ***The author made a number of substantive revisions that improved the paper, including additional simulations and results, clarifications of methodology and figure presentation, and expanded discussion. All of the concerns I addressed in my original comments have been results. Thanks to the authors and the editor for a pleasant and productive review process :)***

AR: We thank the reviewer for these kind words, and for their help in strengthening the paper!

2. Reviewer #2

RC: ***I am grateful for the detailed responses to my comments and for the addition of the policy-based learner, which provides a very informative comparison and interesting new results.***

AR: We are glad that the reviewer finds our addition of the policy-based model helpful.

RC: ***However, the results text provides very little interpretation of the differences between belief-based and policy-based learning, while no mention of this distinction is provided at all in the discussion. Some patterns, such as the lack of maladaptivity in Fig. 3 for the policy-based learning, markedly less negative delta final proportion values in Fig. 2, a larger proportion of conformist learners in Fig. 5, and greater proportion of asocial learners in Fig. 6 deserve some attention in the interpretation of the results.***

AR: This is a fair criticism; it is true that the differences between policy-based and belief-based results were underdiscussed in the revised manuscript. We have added some interpretation of the specific patterns pointed out by the reviewer here to the relevant Results sections; as well as some text to the discussion that highlights what we feel to be the most interesting difference between the two variants (and connects it to the recent work of Roberts-Gaal that was previously pointed out to us by this reviewer). For convenience, we paraphrase the explanations/interpretations here:

- **Lack of maladaptivity for policy-based SL in Figure 3:** This boils down to the fact that belief-based SL can "contaminate" future beliefs (and thus behaviour), while policy-based SL cannot (it is trial-local). If the belief-based social learner obtains incorrect cues, their posterior can be pushed in

the wrong direction—if they would have otherwise been capable of solving the task through asocial learning (i.e. in a low-complexity environment), then this can lead to them performing worse than their asocial-learning peers. By contrast, an incorrect action copied by the policy-based learner will cause them to perform poorly on the current trial, but will have no impact on future trials. Policy-based social learning may therefore be *ineffective*, but if actions are being copied solely from asocial learners, it cannot be actively maladaptive.

- **Weaker negative selection under policy-based SL in Figure 4:** The explanation for this result is similar. The difference between this experiment and the previous one is that now social learners may learn from other social learners (rather than being guaranteed to learn only from asocial learners). This introduces the possibility of a frequency-dependent effect a la Rogers paradox: social learning can become maladaptive if too many agents are doing it, as the supply of "new" (independently obtained) social information is reduced. This is why we now see *some* maladaptivity in the policy-based case; but in addition to this, belief-based SL can still be maladaptive for non-frequency-dependent reasons as in Experiment 1 (e.g. by poisoning learners' beliefs). Thus we see greater negative selection in the belief-based case.
- **More conformist learners under policy-based SL in Figure 5:** The explanation is less obvious here. One plausible interpretation is simply that the difference between the conformist and copy-fittest strategies is sharper in the policy-based model, where the learner directly executes either the majority or fittest agent's action—the impact on behaviour is thus very direct. Each social cue obtained by the belief-based learner still has to be integrated into their posterior (via their own capacity bottleneck), and so its impact on behaviour is less direct, especially under low capacity. The benefits of either strategy may therefore be softened in the belief-based model relative to the policy-based model.
- **More asocial learners under policy-based SL in Figure 6:** This is likely a direct consequence of Rogers-paradox-like dynamics. In the belief-based model, social learners are not pure free-riders; while they use information from others to update their beliefs, they still choose actions and emit their own cues independently. By contrast, policy-based social learners *are* pure free-riders, contributing no independent information back to the common pool. Asocial learners are thus more valuable to the population under policy-based than belief-based social learning; leading us to observe a much stronger frequency-based effect in the former case.

Relatedly, while addressing these points, we found an error in the Figure 3 caption that reversed which panel corresponded to which variant; this is now fixed.

RC: ***Additionally, Line 227 has a broken citation***

AR: This is now fixed.
