# Supplementary material for: Representational capacity shapes both the “whether” and “how” of social learning
Source: PLoS Comput Biol. 2026 Jul 15;22(7):e1014504. doi: 10.1371/journal.pcbi.1014504 (PMC13384400; doi:10.1371/journal.pcbi.1014504)
Supplement: Peer review history

# Peer review: Decision Letter 0

**DOI:** 10.1371/journal.pcbi.1014504.r001

**Authors:** Zhaolei Zhang, Alireza Soltani

14 Apr 2026

PCOMPBIOL-D-26-00658

Cognitive capacity shapes both the “whether” and “how” of social learning

PLOS Computational Biology

Dear Dr. Taylor-Davies,

Thank you for submitting your manuscript to PLOS Computational Biology. After careful consideration, we feel that it has merit but does not fully meet PLOS Computational Biology's publication criteria as it currently stands. Therefore, we invite you to submit a revised version of the manuscript that addresses the points raised during the review process.

Please submit your revised manuscript by Jun 14 2026 11:59PM. If you will need more time than this to complete your revisions, please reply to this message or contact the journal office at ploscompbiol@plos.org. When you're ready to submit your revision, log on to https://www.editorialmanager.com/pcompbiol/ and select the 'Submissions Needing Revision' folder to locate your manuscript file.

Please include the following items when submitting your revised manuscript:

* A letter that responds to each point raised by the editor and reviewer(s). You should upload this letter as a separate file labeled 'Response to Reviewers'. This file does not need to include responses to formatting updates and technical items listed in the 'Journal Requirements' section below.

* A marked-up copy of your manuscript that highlights changes made to the original version. You should upload this as a separate file labeled 'Revised Manuscript with Track Changes'.

* An unmarked version of your revised paper without tracked changes. You should upload this as a separate file labeled 'Manuscript'.

If you would like to make changes to your financial disclosure, competing interests statement, or data availability statement, please make these updates within the submission form at the time of resubmission. Guidelines for resubmitting your figure files are available below the reviewer comments at the end of this letter.

We look forward to receiving your revised manuscript.

Kind regards,

Alireza Soltani

Academic Editor

PLOS Computational Biology

Zhaolei Zhang

Section Editor

PLOS Computational Biology

**Journal Requirements:**

-->1) We ask that a manuscript source file is provided at Revision. Please upload your manuscript file as a .doc, .docx, .rtf or .tex. If you are providing a .tex file, please upload it under the item type u2018LaTeX Source Fileu2019 and leave your .pdf version as the item type u2018Manuscriptu2019.

2) Please provide an Author Summary. This should appear in your manuscript between the Abstract (if applicable) and the Introduction, and should be 150-200 words long. The aim should be to make your findings accessible to a wide audience that includes both scientists and non-scientists. Sample summaries can be found on our website under Submission Guidelines:

https://journals.plos.org/ploscompbiol/s/submission-guidelines#loc-parts-of-a-submission

3) Your manuscript is missing the following sections: Methods.  Please ensure all required sections are present and in the correct order. Make sure section heading levels are clearly indicated in the manuscript text, and limit sub-sections to 3 heading levels. An outline of the required sections can be consulted in our submission guidelines here:

https://journals.plos.org/ploscompbiol/s/submission-guidelines#loc-parts-of-a-submission

4) Please upload all main figures as separate Figure files in .tif or .eps format. For more information about how to convert and format your figure files please see our guidelines:

https://journals.plos.org/ploscompbiol/s/figures

5) We have noticed that you have uploaded Supporting Information files, but you have not included a list of legends. Please add a full list of legends for your Supporting Information files after the references list.

6) Please amend your detailed Financial Disclosure statement. This is published with the article. It must therefore be completed in full sentences and contain the exact wording you wish to be published.

1) State what role the funders took in the study. If the funders had no role in your study, please state: "The funders had no role in study design, data collection and analysis, decision to publish, or preparation of the manuscript.".

7) Your current Financial Disclosure states, "Max Taylor-Davies is supported by the United Kingdom Research and Innovation grant EP/S023208/1, EPSRC Centre for Doctoral Training in Robotics and Autonomous Systems (RAS) ". However, your funding information on the submission form doesn't indicate receiving any funds.Please ensure that the funders and grant numbers match between the Financial Disclosure field and the Funding Information tab in your submission form. Note that the funders must be provided in the same order in both places as well.

8) Some material included in your submission may be copyrighted. According to PLOS’s copyright policy, authors who use figures or other material (e.g., graphics, clipart, maps) from another author or copyright holder must demonstrate or obtain permission to publish this material under the Creative Commons Attribution 4.0 International (CC BY 4.0) License used by PLOS journals. Please closely review the details of PLOS’s copyright requirements here: PLOS Licenses and Copyright. If you need to request permissions from a copyright holder, you may use PLOS's Copyright Content Permission form.

Please respond directly to this email and provide any known details concerning your material's license terms and permissions required for reuse, even if you have not yet obtained copyright permissions or are unsure of your material's copyright compatibility. Once you have responded and addressed all other outstanding technical requirements, you may resubmit your manuscript within Editorial Manager.

Potential Copyright Issues:

-->Figure 1. Please confirm whether you drew the images / clip-art within the figure panels by hand. If you did not draw the images, please provide (a) a link to the source of the images or icons and their license / terms of use; or (b) written permission from the copyright holder to publish the images or icons under our CC BY 4.0 license. Alternatively, you may replace the images with open source alternatives. See these open source resources you may use to replace images / clip-art:

- https://commons.wikimedia.org

- https://openclipart.org/                                             .

Note: If the reviewer comments include a recommendation to cite specific previously published works, please review and evaluate these publications to determine whether they are relevant and should be cited. There is no requirement to cite these works unless the editor has indicated otherwise.

**Reviewers' comments:**

Reviewer's Responses to Questions

Reviewer #1: This paper presents a series of simulation experiments investigating the effects of cognitive capacity and social learning strategy on the evolution of agents in a simple learning and foraging task. In the task, agents receive partial information about the environment and the preferences of other agents, and decide between eating or discarding the current mushroom. An underlying rule determines the reward outcomes based on N binary features of the mushroom. Agent fitness is tracked: agents die if they fall below a fitness threshold (or periodically with some probability), and are replaced probabilistically with high-fitness agents. In a series of four simulated experiments, the authors investigate the relative abundance of learning strategies within the population as a function of time and “cognitive capacity”, which quantifies agent’s ability to retain information gathered from the environment in their subsequent learning and decision making.

The authors show that both cognitive capacity and social learning strategy have strong effects on the evolution of the population. They begin by comparing asocial learning to unbiased social learning, in which agents randomly query other agents to acquire additional social information. Even in this simple case, where only one social learner is present amongst a population of asocial learners, they show an unexpected non-monotonic relationship between capacity and the benefits of social information use. In subsequent experiments, the authors expand on these findings by stimulating additional evolutionary dynamics, a wider range of social strategies, and more detailed analyses about the resulting population breakdown and/or social exchange. They conclude by drawing several insights about the value of social exchange relative to the complexity of the environment, and how the relative effectiveness of social learning strategies shifts over time and environmental parameters.

I thought this paper was clear, concise, and persuasive. The authors positioned their contribution effectively within the broader field of social learning, arguing that previous studies have failed to look at agent’s information processing capacity and of symmetric constraints in both learners and providers of information. While I am not familiar with these fields, both of these features seem highly relevant in studying social learning in any naturalistic task or when dealing with any realistic cognitive system. The methods section was fairly straightforward, and included the key details necessary to understand the simulated agents and experiments, with only a few points of confusion. Similarly, the four experiments presented in the paper followed a logical progression, from establishing a baseline of behavior and analysis, through the core experiments establishing the U-shaped dependence on capacity, and ending in additional analyses that supported their explanations for the emergence and dominance of certain social learning strategies. The figures were clearly laid out, using colors to effectively communicate a variety of complex relationships. I appreciated the two choices of ‘complex’ social learning strategies, but felt that the intuition and mechanics of these agents could have been elaborated in greater detail (see below). I found the relative success of the four agent types to be one of the most interesting aspects of the paper, and the additional analyses in Fig 5C and 6 seemed to support the author's story about the added benefits of social information in each case. The inverse-U shaped relationships are also interesting, and showcase the utility of using computational modelling to investigate multi-agent social systems, where intuitions can often fail.

I had a few minor complaints and suggestions, which I will list here

The term “cognitive capacity” strikes me as too general, and risks overinflating the (real) value of the paper. The authors clearly note that “There are multiple ways we could achieve this within the TS framework, and we report results for different variants in the SI.” I appreciate this anticipation and engagement with the question, and the SI results do offer some additional insights into how cognitive capacity might be realized in this framework. The SI results also show that the main results hold under these different realizations, although with some variations that might be worth exploring in greater detail. Furthermore, the authors provide citations of similar work that “uses information bottleneck as a principled way to model capacity limits”. Nonetheless, as a cognitive scientist who studies the ability of simulated biological systems to implement abstract cognitive algorithms (like the ones employed here), I still feel that “cognitive capacity” is too big an umbrella for the manipulations being done here. I would be more comfortable with a term like “representational capacity”, which emphasizes that the limitations are on how much information the agents can store (or use) accurately, rather than how they can use it. Representational capacity applies both to zeroing out individual features (the main manipulation), to noise in feedback (SI 1.2), and, to a lesser extent, to imperfect memory (SI 1.1). Representational capacity can be contrasted with “functional capacity”, which emphasises the inability for an agent to compute certain classes of functions entirely. An example of functional capacity might be limitations on agent’s rules for forming or updating belief distributions, their ability to identify the fittest individual, etc. Whatever language the authors decide to use, I would advise them to make limited claims about how broadly their manipulations capture cognitive capacity in intelligent animals. They might also devote some space in the discussion to contextualizing this choice relative to broader notions of social intelligence and the complexity of social exchange.

Similarly, I’d appreciate a bit more qualitative discussion about the choice to “model social learning as an additional belief update … that takes place before choice”, and the choice to model social cues as an expressed belief about edibility. For example, how might the experiments and results change if agents communicated their underlying feature-based belief structures directly?

Equations 8 and 9 for the conformist and fittest social learners need additional elaboration. A few sentences to describe the high-level logic of those learning strategies, and why the particular mathematical forms were chosen to implement this logic, would go a long way.

Fig 5a, why do conformist and copy-fittest make up such a small proportion of the final population across a wide range of capacities? Given Fig 5C, shouldn’t these two strategies generally acquire more correct social information, and subsequently out-compete the naive or asocial learners? This is also the trend I expected given Fig 6B: unbiased learners and asocial learners tend to die out over time. Fig 5A thus seems to contradict one of the central conclusions “Specifically, we found that learners who selectively use cues from only the fittest agents dominated lower-capacity populations”. Perhaps the n=4 environment was an outliner in this case?

Overall, this is an excellent paper that I can happily endorse for publication, with only a few minor edits for clarity and contextualization.

Reviewer #2: This manuscript presents what I found to be a very interesting investigation of how cognitive capacity influences social learning. Through a series of simulations, the author presents findings showing how cognitive capacities influence the emergence of social learning strategies, which can shift from success-biased to conformity-biased depending on capacity. I think these findings are of great interest to a wide readership, but in particular for the field of cultural evolution, which is increasingly interested in more cognitively sophisticated models of social learning.

Overall, I found the paper to be quite well written, although I have a number of minor clarification issues I address at the bottom. In addition, I also identified some relatively more major issues, which I address now in more detail.

1. Cognitive costs of social learning

Here the authors focus on a setting where cognitive capacity only influences asocial learning. However, different kinds of social learning also come with their associated cognitive costs (Wu, Velez & Cushman, 2022). Simple imitation may be quite cheap (as is assumed in these simulations), but more sophisticated forms of social learning, such as Theory of Mind inference, are arguably even more complex than asocial learning. Thus, decreasing capacity doesn’t necessarily favor social learning, since social learning also comes with cognitive costs. I think this could be clarified in the paper.

Additionally, I think there are some idiosyncrasies in how social learning is implemented here that need to be unpacked in greater detail. Line 262 states that the focus is on the initial emergence (in evolutionary timescales) of social information, which is used to justify an initial focus on indiscriminate social learning. However, if that is the case, why do you need the rather complex Eq. 6 whereby an expectation of reward is emitted by each peer, which is then binarized in a somewhat arbitrary fashion. Wouldn’t it be more straightforward to just implement frequency dependent copying? Or some form of policy-based social learning rather than a more complex value-based strategy (cf. hierarchy from Wu, Velez & Cushman, 2022)? Surely biased social learning would emerge before the kind of social learning represented in Eq. 6? Perhaps a better justification could be provided or the limitations of these assumptions mentioned more explicitly in the discussion.

2. Cognitive capacity via corrupting reward encodings vs. effortful uncertainty directed explanation.

I thought the current implementation of capacity was quite innovative (Eq. 4). However, I also found there to be rather limited connections to previous work in resource rational (asocial) learning. The Lai & Gershman work is applied to policy complexity and Zaslavsky applies it to naming conventions. Of course it’s justifiable to introduce this as a new mechanism, however it also feels like a missed opportunity to connect to more well-studied mechanisms of asocial learning that are associated with cognitive costs. For instance, by connecting to directed exploration as being an aspect of asocial learning is specifically sensitive to reduced cognitive capacity, be it from working memory load (Cogliati-Dezza, Cleeremans, & Alexander, 2019) or time pressure (Wu, Schulz, Pleskac & Speekenbrink, 2022). This could be implemented using UCB sampling, where more limited agents would have lower information bonuses for exploration. I’m not insisting on this also being added to the simulations, but some connection to the cognitive costs of exploration could help this work reach a broader audience.

3. Difficulty in interpreting the figures.

I found some of the results very hard to read from the figures. This was particularly the case for Figure 4, where even after spending a lot of time with it, I still don’t think I really understand what the red line and the green bars represent. In addition, plots showing related results present them in entirely different formats, making it hard to compare findings from one set of simulations to the next.

Additionally, I was also wondering why no strategy ever dominates in the simulations. Is this potentially suggesting they haven’t been run for enough iterations? What implications would there be for this lack of dominance of a single strategy? Would we expect a proportion of the population to be incapable of social learning? Or would we expect some degree of specialization?

4. Minor comments

- Line 33-34 “critical mass”. Isn’t the issue usually the opposite? There’s high frequency dependent fitness for being a social learner when the population is full of asocial learners, but low fitness when the population is saturated with social learners (Rogers, 1988). Thus, it’s easy for social learning to invade, but difficult for it to dominate.

- Line 83: Barkoczi & Galesic (2018) address this question. Although it’s still true it’s been less studied

- Fig. 1. More labels are needed since the figures are not very self-explanatory. Panel c needs axis labels and the n variable should perhaps be explained

- Line 118. This seems similar to the findings from Roberts-Gaal, Bolic, & Cushman (2025)

- line 124. There are multiple definitions of contextual bandit. Maybe cite a similar paradigm for clarity.

- Reward function. The manuscript uses a rule-based reward function. What idiosyncrasies of rule-based generalization are being captured here vs. alternative accounts of similarity-based generalization (see Wu, Meder & Schulz, 2025 for a recent review)? Would this potentially influence the social vs. asocial trade-off? Perhaps this could be mentioned in the discussion

- Line 172. Perhaps clarify that this is the asocial strategy

- Line 184 is separated by an entire paragraph before the related equation at the top of Eq. 4 is presented. This maybe it a bit hard to follow the methods

- line 228. Why is this the case? Is it because of noisy exploration induced by noisy representations?

- Fig. 3 please use a continuous color scale for n to make it easier to read. Also try to avoid a stroop effect where some of the n-colors also accidentally align with the top and lower part of the figure (adaptive vs. maladaptive) that may mislead the reader

- Line 338. Why does it disappear?

- Line 353. How can we verify this? These are entirely different figures

- Fig. 5. Why does no strategy dominate? Is this a potential

I sign the review to please excuse any biases in mentioning my own papers or those in my more immediate circles, since they’re what I’m most familiar with. I have no expectation that you include these citations in a revised version of the manuscript, especially when they are not relevant.

- Charley M. Wu

**********

**Have the authors made all data and (if applicable) computational code underlying the findings in their manuscript fully available?**

The PLOS Data policy requires authors to make all data and code underlying the findings described in their manuscript fully available without restriction, with rare exception (please refer to the Data Availability Statement in the manuscript PDF file). The data and code should be provided as part of the manuscript or its supporting information, or deposited to a public repository. For example, in addition to summary statistics, the data points behind means, medians and variance measures should be available. If there are restrictions on publicly sharing data or code —e.g. participant privacy or use of data from a third party—those must be specified.

Reviewer #1: Yes

Reviewer #2: Yes

**********

PLOS authors have the option to publish the peer review history of their article (what does this mean?). If published, this will include your full peer review and any attached files.

If you choose “no”, your identity will remain anonymous but your review may still be made public.

**Do you want your identity to be public for this peer review?**  For information about this choice, including consent withdrawal, please see our Privacy Policy.

Reviewer #1: **Yes:** Peter Duggins

Reviewer #2: **Yes:** Charley M. Wu

[NOTE: If reviewer comments were submitted as an attachment file, they will be attached to this email and accessible via the submission site. Please log into your account, locate the manuscript record, and check for the action link "View Attachments". If this link does not appear, there are no attachment files.]

**Figure resubmission:**

-->While revising your submission, we strongly recommend that you use PLOS’s NAAS tool (https://ngplosjournals.pagemajik.ai/artanalysis) to test your figure files. NAAS can convert your figure files to the TIFF file type and meet basic requirements (such as print size, resolution), or provide you with a report on issues that do not meet our requirements and that NAAS cannot fix.

After uploading your figures to PLOS’s NAAS tool - https://ngplosjournals.pagemajik.ai/artanalysis, NAAS will process the files provided and display the results in the "Uploaded Files" section of the page as the processing is complete. If the uploaded figures meet our requirements (or NAAS is able to fix the files to meet our requirements), the figure will be marked as "fixed" above. If NAAS is unable to fix the files, a red "failed" label will appear above. When NAAS has confirmed that the figure files meet our requirements, please download the file via the download option, and include these NAAS processed figure files when submitting your revised manuscript.

**Reproducibility:**

To enhance the reproducibility of your results, we recommend that authors of applicable studies deposit laboratory protocols in protocols.io, where a protocol can be assigned its own identifier (DOI) such that it can be cited independently in the future. Additionally, PLOS ONE offers an option to publish peer-reviewed clinical study protocols. Read more information on sharing protocols at https://plos.org/protocols?utm_medium=editorial-email&utm_source=authorletters&utm_campaign=protocols

# Author comment: Author response to Decision Letter 1

**DOI:** 10.1371/journal.pcbi.1014504.r002

27 May 2026

Attachment Submitted filename: response-letter.pdf

# Peer review: Decision Letter 1

**DOI:** 10.1371/journal.pcbi.1014504.r003

**Authors:** Zhaolei Zhang, Alireza Soltani

16 Jun 2026

PCOMPBIOL-D-26-00658R1

Representational capacity shapes both the “whether” and “how” of social learning

PLOS Computational Biology

Dear Dr. Taylor-Davies,

Thank you for submitting your manuscript to PLOS Computational Biology. After careful consideration, we feel that it has merit but does not fully meet PLOS Computational Biology's publication criteria as it currently stands. Therefore, we invite you to submit a revised version of the manuscript that addresses the issues raised during the review process. In particular, we agree with Reviewer #2 that some of the new analyses should be better integrated into the overall narrative and discussed more thoroughly. **Please note that the revised manuscript will not be sent for external review; instead, the final decision will be made at the editorial level.**

Please submit your revised manuscript by Aug 16 2026 11:59PM. If you will need more time than this to complete your revisions, please reply to this message or contact the journal office at ploscompbiol@plos.org. When you're ready to submit your revision, log on to https://www.editorialmanager.com/pcompbiol/ and select the 'Submissions Needing Revision' folder to locate your manuscript file.

Please include the following items when submitting your revised manuscript:

* A letter that responds to each point raised by the editor and reviewer(s). You should upload this letter as a separate file labeled 'Response to Reviewers'. This file does not need to include responses to formatting updates and technical items listed in the 'Journal Requirements' section below.

* A marked-up copy of your manuscript that highlights changes made to the original version. You should upload this as a separate file labeled 'Revised Manuscript with Track Changes'.

* An unmarked version of your revised paper without tracked changes. You should upload this as a separate file labeled 'Manuscript'.

If you would like to make changes to your financial disclosure, competing interests statement, or data availability statement, please make these updates within the submission form at the time of resubmission. Guidelines for resubmitting your figure files are available below the reviewer comments at the end of this letter.

As the corresponding author, your ORCID iD is verified in the submission system and will appear in the published article. PLOS supports the use of ORCID, and we encourage all coauthors to register for an ORCID iD and use it as well. Please encourage your coauthors to verify their ORCID iD within the submission system before final acceptance, as unverified ORCID iDs will not appear in the published article. Only the individual author can complete the verification step; PLOS staff cannot verify ORCID iDs on behalf of authors.

We look forward to receiving your revised manuscript.

Kind regards,

Alireza Soltani

Academic Editor

PLOS Computational Biology

Zhaolei Zhang

Section Editor

PLOS Computational Biology

**Additional Editor Comments (if provided):**

**Journal Requirements:**

If the reviewer comments include a recommendation to cite specific previously published works, please review and evaluate these publications to determine whether they are relevant and should be cited. There is no requirement to cite these works unless the editor has indicated otherwise.

1) The uploaded Manuscript file contains highlights/ tracked changes. Please provide a clean version of your manuscript from your latest manuscript version.

2) Please upload a copy of Figures 1A-C, 2, 3AB, 4A-D, 5A-F, and 6A-F which you refer to in your text on pages 3, 4, 5, 7, and 8. Or, if the figure is no longer to be included as part of the submission please remove all reference to it within the text.

3) We have noticed that you have uploaded Supporting Information files, but you have not included a list of legends. Please add a full list of legends for your Supporting Information files after the references list.

**Reviewers' comments:**

Reviewer's Responses to Questions

**Comments to the Authors:**

**Please note here if the review is uploaded as an attachment.**

Reviewer #1: The author made a number of substantive revisions that improved the paper, including additional simulations and results, clarifications of methodology and figure presentation, and expanded discussion. All of the concerns I addressed in my original comments have been results. Thanks to the authors and the editor for a pleasant and productive review process :)

Peter Duggins

Reviewer #2: I am grateful for the detailed responses to my comments and for the addition of the policy-based learner, which provides a very informative comparison and interesting new results.

However, the results text provides very little interpretation of the differences between belief-based and policy-based learning, while no mention of this distinction is provided at all in the discussion. Some patterns, such as the lack of maladaptivity in Fig. 3 for the policy-based learning, markedly less negative \delta final proportion values in Fig. 2, a larger proportion of conformist learners in Fig. 5, and greater proportion of asocial learners in Fig. 6 deserve some attention in the interpretation of the results.

Additionally, Line 227 has a broken citation

**********

**Have the authors made all data and (if applicable) computational code underlying the findings in their manuscript fully available?**

The PLOS Data policy requires authors to make all data and code underlying the findings described in their manuscript fully available without restriction, with rare exception (please refer to the Data Availability Statement in the manuscript PDF file). The data and code should be provided as part of the manuscript or its supporting information, or deposited to a public repository. For example, in addition to summary statistics, the data points behind means, medians and variance measures should be available. If there are restrictions on publicly sharing data or code —e.g. participant privacy or use of data from a third party—those must be specified.

Reviewer #1: Yes

Reviewer #2: Yes

**********

PLOS authors have the option to publish the peer review history of their article (what does this mean?). If published, this will include your full peer review and any attached files.

If you choose “no”, your identity will remain anonymous but your review may still be made public.

**Do you want your identity to be public for this peer review?**  For information about this choice, including consent withdrawal, please see our Privacy Policy.

Reviewer #1: **Yes:** Peter Duggins

Reviewer #2: No

[NOTE: If reviewer comments were submitted as an attachment file, they will be attached to this email and accessible via the submission site. Please log into your account, locate the manuscript record, and check for the action link "View Attachments". If this link does not appear, there are no attachment files.]

**Figure resubmission:**

-->While revising your submission, we strongly recommend that you use PLOS’s NAAS tool (https://ngplosjournals.pagemajik.ai/artanalysis) to test your figure files. NAAS can convert your figure files to the TIFF file type and meet basic requirements (such as print size, resolution), or provide you with a report on issues that do not meet our requirements and that NAAS cannot fix.

After uploading your figures to PLOS’s NAAS tool - https://ngplosjournals.pagemajik.ai/artanalysis, NAAS will process the files provided and display the results in the "Uploaded Files" section of the page as the processing is complete. If the uploaded figures meet our requirements (or NAAS is able to fix the files to meet our requirements), the figure will be marked as "fixed" above. If NAAS is unable to fix the files, a red "failed" label will appear above. When NAAS has confirmed that the figure files meet our requirements, please download the file via the download option, and include these NAAS processed figure files when submitting your revised manuscript.

**Reproducibility:**

To enhance the reproducibility of your results, we recommend that authors of applicable studies deposit laboratory protocols in protocols.io, where a protocol can be assigned its own identifier (DOI) such that it can be cited independently in the future. Additionally, PLOS ONE offers an option to publish peer-reviewed clinical study protocols. Read more information on sharing protocols at https://plos.org/protocols?utm_medium=editorial-email&utm_source=authorletters&utm_campaign=protocols

# Author comment: Author response to Decision Letter 2

**DOI:** 10.1371/journal.pcbi.1014504.r004

25 Jun 2026

Attachment Submitted filename: response-letter_auresp_2.pdf

# Editor report: Decision Letter 2

**DOI:** 10.1371/journal.pcbi.1014504.r005

**Authors:** Zhaolei Zhang, Alireza Soltani

27 Jun 2026

Dear Mr Taylor-Davies,

We are pleased to inform you that your manuscript 'Representational capacity shapes both the “whether” and “how” of social learning' has been provisionally accepted for publication in PLOS Computational Biology.

Before your manuscript can be formally accepted you will need to complete some formatting changes, which you will receive in a follow up email. A member of our team will be in touch with a set of requests.

Please note that your manuscript will not be scheduled for publication until you have made the required changes, so a swift response is appreciated.

IMPORTANT: The editorial review process is now complete. PLOS will only permit corrections to spelling, formatting or significant scientific errors from this point onwards. Requests for major changes, or any which affect the scientific understanding of your work, will cause delays to the publication date of your manuscript.

Should you, your institution's press office or the journal office choose to press release your paper, you will automatically be opted out of early publication. We ask that you notify us now if you or your institution is planning to press release the article. All press must be co-ordinated with PLOS.

Thank you again for supporting Open Access publishing; we are looking forward to publishing your work in PLOS Computational Biology.

Best regards,

Alireza Soltani

Academic Editor

PLOS Computational Biology

Zhaolei Zhang

Section Editor

PLOS Computational Biology

***********************************************************

# Editor report: Acceptance letter

**DOI:** 10.1371/journal.pcbi.1014504.r006

**Authors:** Zhaolei Zhang, Alireza Soltani

PCOMPBIOL-D-26-00658R2

Representational capacity shapes both the “whether” and “how” of social learning

Dear Dr Taylor-Davies,

I am pleased to inform you that your manuscript has been formally accepted for publication in PLOS Computational Biology. Your manuscript is now with our production department and you will be notified of the publication date in due course.

The corresponding author will soon be receiving a typeset proof for review, to ensure errors have not been introduced during production. Please review the PDF proof of your manuscript carefully, as this is the last chance to correct any errors. Please note that major changes, or those which affect the scientific understanding of the work, will likely cause delays to the publication date of your manuscript.

Soon after your final files are uploaded, unless you have opted out, the early version of your manuscript will be published online. The date of the early version will be your article's publication date. The final article will be published to the same URL, and all versions of the paper will be accessible to readers.

For Research, Software, and Methods articles, you will receive an invoice from PLOS for your publication fee after your manuscript has reached the completed accept phase. If you receive an email requesting payment before acceptance or for any other service, this may be a phishing scheme. Learn how to identify phishing emails and protect your accounts at https://explore.plos.org/phishing.

Thank you again for supporting PLOS Computational Biology and open-access publishing. We are looking forward to publishing your work!

With kind regards,

Janani Seenivasan

PLOS Computational Biology | Carlyle House, Carlyle Road, Cambridge CB4 3DN | United Kingdom ploscompbiol@plos.org | Phone +44 (0) 1223-442824 | ploscompbiol.org | @PLOSCompBiol
